# Use of interferon gamma-based assay to diagnose tuberculosis infection in health care workers after short term exposure

**DOI:** 10.1186/1471-2334-9-60

**Published:** 2009-05-11

**Authors:** Dag Gundersen Storla, Ingun Kristiansen, Fredrik Oftung, Gro Ellen Korsvold, Monica Gaupset, Gerd Gran, Anne Kristin Øverby, Anne Margarita Dyrhol-Riise, Gunnar Aksel Bjune

**Affiliations:** 1 Department of International Health, Institute of General Practice and Community Medicine, University of Oslo, PO Box 1130 Blindern, N-0318 Oslo, Norway; 2 Center for Health Promotion, Akershus University Hospital, Sykehusveien 27, N-1478 Lørenskog, Norway; 3 Division of Infectious Disease Control, Norwegian Institute of Public Health, P.O. Box 4404 Nydalen, N-0403 Oslo, Norway; 4 Competence Centre for Imported and Tropical Diseases, Ullevål University Hospital, Kirkeveien 166, N-0450 Oslo, Norway; 5 Department of Medicine, Haukeland University Hospital, Jonas Lies vei 65, N-5053 Bergen, Norway; 6 Tuberculosis Control Unit, Ullevål University Hospital, Kirkeveien 166, N-0450 Oslo, Norway

## Abstract

**Background:**

We intended to assess the risk for health care workers (HCWs) of acquiring *M. tuberculosis *infection after exposure to patients with sputum-smear positive pulmonary tuberculosis at three University Hospitals (Ullevål, Akershus, and Haukeland) in Norway.

**Methods:**

We tested 155 exposed health care workers and 48 healthy controls both with a tuberculin skin test (Mantoux) and the T-SPOT.*TB *test, a recently developed interferon-γ release assays based on the *M. tuberculosis*-specific ESAT-6 and CFP10 antigens, to investigate if this test might improve infection control measures.

**Results:**

Among the 155 exposed HCWs tested in this study, 27 individuals were defined as newly infected cases by TST after recent exposure, while only 3 of these had a positive T-SPOT.*TB *test. The number of T-SPOT.*TB *positives represents 11% of the individuals defined as recently infected by TST after exposure (3/27) and 2% of the total number of exposed people tested (3/155). In addition, 15 individuals had been previously defined as infected by TST before exposure of whom 2 subjects were T-SPOT.*TB *positive. All individuals detected as T-SPOT.*TB *positive belonged to the TST positive group (> 15 mm), and the percentage concordance between T-SPOT.*TB *and TST, including both previously and newly infected subjects, was 12% (5/42). The 48 control participants used in the study were all T-SPOT.*TB *negative, but 3 of these subjects were TST positive.

**Conclusion:**

Our data indicate that the frequency of latent TB in the total cohort of HCWs is 3%, whereas the rate of transmission of TB to exposed individuals is approximately 2% and occurs through exposure periods of short duration. Thus, the risk of TB transmission to HCWs following TB exposure in a hospital setting in Norway is low, and improved screening approaches will benefit from the application of specific interferon-γ release assays.

## Background

In Norway the incidence of tuberculosis (TB) is generally low, and surveillance is primarily based on the detection and treatment of latent infection in risk groups and contact tracing following exposure. It is well established that most of the infected individuals will not progress to active disease, but will maintain a latent infection. Although a latent infection is clinically silent and not contagious, it can reactivate to cause highly contagious pulmonary tuberculosis, the most prevalent form of the disease in adults [1]. Several studies have concluded that transmission is usually caused by prolonged contact with an infectious case of TB, and the risk of being infected is dependent upon the amount of time spent sharing room air with the index case [2]. Still, contact tracing utilizing the fingerprinting methods IS6110-restriction fragment length polymorphism (RFLP) and spoligotyping indicate that a substantial proportion of TB cases acquire infection as a result of casual exposures of short duration [3]. Health care workers are constantly at risk of exposure, and reliable and specific diagnostic tools are essential to improve follow-up procedures. As most new infections result in latent TB infection, strain fingerprinting can not be used to trace transmission.

The current policy in Norwegian health institutions is to perform environmental screenings based on the Mantoux Tuberculin Skin Test (TST) after TB exposure. According to international guidelines, individuals with a TST > 5 mm in non-vaccinated persons and TST > 10 mm in persons vaccinated with Bacillus Calmette-Guérin (BCG) are considered to be infected with *M. tuberculosis*. In Norway, BCG vaccination is routinely offered to TST-negative children at the age of 14 years. In addition, it has been compulsory for all health care workers to be vaccinated with BCG if a positive TST has not been documented. Thus, in Norway the TST status of health care workers is usually known and the post-exposure TST is compared to the pre-exposure TST based on the increase in the size of the induration. In accordance with the national guidelines, the definition of post-exposure infection is an increase in TST induration of ≥ 10 mm or a TST of ≥ 15 mm if previous TST status is un-known [4]. Norwegian hospital personnel defined as infected are followed up with consultations and annual chest x-rays for three years. In addition, if an employee is likely infected, prophylactic treatment is offered. A major problem in many hospitals is that the same personnel have undergone multiple screenings, which result in boosting of their TST response. Thus, due to both low specificity and TST-induced boosting [5], a large proportion of the group defined as infected after exposure are probably false positives. This may lead to incorrect treatment, waste of resources, and unnecessary anxiety.

However, recently developed interferon-γ release assays (IGRAs), based on the *Mtb*-specific antigens ESAT-6 and CFP10, have contributed to improved specificity in TB screening. These RD1-encoded protein antigens are absent from all vaccine strains of *M. bovis *BCG and most non-tuberculous mycobacteria (except *M. marinum, M. szulgai, M. Kansasii*). Such tests can therefore distinguish *Mtb *infection from infections caused by other mycobacteria or previous BCG vaccination [5,6]. Two commercially available and regulatory agency approved test systems can be used: T-SPOT.*TB *(Oxford Immunotec) is an ELISPOT assay based on the analysis of a defined number of isolated peripheral blood mononuclear cells, whereas Quantiferon-TB Gold (QFT) (Cellestis) is a whole blood in-tube ELISA-based test. The tests have comparable specificity (98–99%), but sensitivity is reported to be somewhat higher for the T-SPOT.*TB *test (97%) compared to the QFT assay (90%). This difference is primarily pronounced in immuno-suppressed persons and in children, where the frequency of indeterminate results is demonstrated to be higher for the QFT assay [7,8].

The specificity of IFN-γ release assays has the potential to improve both the diagnosis of TB in infected individuals and the utilisation of public health resources for TB control. In this study, we have compared the T-SPOT.*TB *test with TST in hospital personnel exposed to TB. We have used the results to assess the role of IFN-γ release assays for improved screening of this target group, and determine the rate of TB transmission during short exposure in a TB low-endemic country.

## Methods

### Study groups and design

From March 2005 to January 2007, 155 TB-exposed health care workers were included from three major University Hospitals in Norway: Haukeland University Hospital (HUS), Ullevål University Hospital (UUS), and Akershus University Hospital (AHUS). For inclusion in the study, exposed persons had to be in close contact (stay in the same room) with a sputum-smear positive pulmonary TB patient in a non-protected manner for at least 1 hour. Subjects were then grouped according to the time of exposure: 'Low exposure' was defined as 1 to 8 cumulative hours of close contact, while more than 8 cumulative hours of close contact was considered to be 'high exposure'.

A control group of 48 non-exposed individuals were recruited from the non-clinical staff at AHUS. Only individuals without any known prior exposure where included in this group. The epidemiological data are summarized in Table 1. The mean age of the control group was 41 years, with a female:male ratio of 33:15. The exposed group was a mean age of 39 years, with a female:male ratio of 132:22. Ten employees came from TB high-endemic countries. Both groups lived in the same geographical area and consisted of employees at Norwegian hospitals with middle incomes. The exposed group was followed up according to the national guidelines, including TST and chest X-ray independent of the T-SPOT.*TB *test result [4]. All participants answered a questionnaire concerning BCG vaccination status, former TB, previous exposure, and residency in TB high-endemic countries. Previous studies have demonstrated that individuals infected with TB complete their cellular immune response within 8 weeks after exposure [9]. Thus, both the T-SPOT.*TB *test and the TST were performed as close to 8 weeks after exposure as possible (mean, 11.5 weeks). The subjects had not been tested previously by IFN-γ test, so their pre-exposure T-SPOT.*TB *status was not known. The last documented TST found in the hospital records and TST results obtained 8 weeks after exposure were used as the basis for determining infection status. The study was approved by the Regional Committee for Medical Research Ethics East (REK Øst). Informed consent was obtained from all participants, clarifying that follow up and treatment would be offered regardless of participation and according to national guidelines [4].

**Table 1:** Infection status versus age, sex, migration, exposure time, and Mantoux status

Characteristics	New TST positives HCWs (n = 27)	Previously defined as TST positive HCWs (n = 15)	TST negatives HCWs (n = 113)	**Healthy Controls** (n = 48)
Age (years, mean (range))	38.9 (22–65)	38.9 (22–65)	38.9 (22–65)	41.4 (20–67)
Sex (female/male)	22/5	12/3	98/15	33/15
Migration				
1. Born in a low-endemic area	25	12	101	48
2. Worked in a high-endemic area > 6 months	0	0	6	
3. Born in a high-endemic area	2	2	6	
Exposure time (≤ 8 h/> 8 h)	20/7	10/4	84/29	
Mantoux (mm) before exposure (median)	8		6/13	5/4
Mantoux (mm) after exposure (median)	19		6/6	
Vesiculous Mantoux after exposure	8			

*Results in absolute numbers from post-exposure screening of 155 health care workers (HCWs) and 48 healthy controls, 2005–2007. During childhood all participants were either BCG-immunized or had a naturally positive tuberculin skin test (TST). The definition of a positive TST (Mantoux) was an increase of ≥ 10 mm, or of ≥ 15 mm if previous TST status was unknown (in concordance with the national guidelines). Some HCWs had been defined as TST positive during previous post-exposure screenings (15), while others were defined as infected during the current study (27).

### Tuberculin skin test

TSTs of both exposed personnel and controls were performed according to the Mantoux method with Purified Protein Derivate (PPD) RT 23 SSI, (2 TU) from SSI, Copenhagen, Denmark. Transverse induration in mm at the injection site was measured after 48–72 hours, and the results were interpreted according to the national guidelines [4]. Reading of test results was repeated if the induration was large, showed signs of adverse reactions, or was difficult to read.

### T-SPOT.*TB *test

The T-SPOT.*TB *test, (Oxford Immunotec, UK), was used according to the manufacturer's instructions. The blood was always drawn prior to the TST to avoid any possible interactions caused by rapid homing of specific T-lymphocytes to the tuberculin injection site. Venous blood drawn into Cell Preparation Tube (CPT) vacutainers (Beckton Dickinson, Oxford, UK) was sent by courier service and analysed within 6 hours. In brief, peripheral blood mononuclear cells (PBMC) were isolated from blood following centrifugation, washed and counted. PBMCs at a concentration of 250,000 cells/well in AIM V^® ^cell culture medium (Invitrogen Corporation, Carlsbad, USA) were stimulated with ESAT-6 and CFP10 in 96-well plates pre-coated with anti-IFN-γ capture antibodies, and incubated overnight at 37°C in 5% CO_2_. Medium only and mitogen (Phytohemagglutinin) were used as negative and positive controls, respectively. The next day the T-SPOT.*TB *assay was developed by adding an alkaline phosphatase-conjugated detection antibody and substrate. Coloured spots, representing individual INF-γ-producing T cells, were counted manually using a microscope. The results were recorded based on the definition of positive and negative reactions given in the instructions from the manufacturer. All initial positive results were confirmed by analysis of a second blood sample before they were reported as positive.

### Data handling and statistical analysis

All data were entered into a central Microsoft Access™ database, approved by the Norwegian Data Inspectorate, and statistical analysis was performed with the Graph Pad Prism 4™ software.

## Results

Among the 155 exposed health care workers tested, 27 individuals were defined as newly infected cases by TST after recent exposure, while only 3 of these had a positive T-SPOT.*TB *test (Table 2 and 3). There were no indeterminate test results, and no excluded HCWs or T-SPOT.*TB *results. The number of T-SPOT.*TB *positives represents 11% of the individuals defined as recently infected by TST after exposure (3/27) and 2% of the total number of exposed people tested (3/155). In addition, 15 individuals had been previously defined as infected by TST before exposure of whom 2 were T-SPOT.*TB *positive: one was born in a TB high-endemic country, and the other had been previously treated for pulmonary TB. Interestingly, the former had a negative TST at the time of immigration to Norway, raising a question concerning the source and place of infection.

**Table 2:** Concordance between TST and T-SPOT.TB Results

Characteristics	New TST positives	Previously defined as TST positives	Total
TST positives among exposed (%)	27/155 (17%)	15/155 (10%)	42/155 (27%)
T-SPOT.*TB ***-**positives among exposed (%)	3/155 (2%)	2/155 (1%)	5/155 (3%)
T-SPOT.*TB ***-**positives among TST positives (%)	3/27 (11%)	2/15 (13%)	5/42 (12%)

Table 2 depicts concordance between tuberculin skin test (TST) and T-SPOT.TB results. Results in absolute numbers (percentages in brackets) from post-exposure screening of 155 health care workers (HCWs) and 48 healthy controls, 2005–2007. During childhood all participants were either BCG-immunized or had a naturally positive TST. The definition of a positive TST (Mantoux) was an increase of ≥ 10 mm, or of ≥ 15 mm if previous TST status was unknown (in concordance with the national guidelines). The exposed HCWs were also tested with T-SPOT.*TB*, an interferon-gamma release assay (IGRA). Some HCWs had been defined as TST positives during previous post-exposure screenings (15) while others were defined as TST positives during the current study (27).

**Table 3:** Characteristics of the 5 T-SPOT.TB-positive HCWs

Characteristics	HCW 1	HCW 2	HCW 3	HCW 4	HCW 5
Mantoux (mm) median pre/post exposure	Previously TST positive	12 mm and vesicular	16 mm and vesicular	Previous tuberculosis	15 mm and vesicular
TST positive	Not tested	Yes	Yes	Not tested	Yes
Exposure time (≤ 8 h/> 8 h)	≤ 8 h	≤ 8 h	≤ 8 h	≤ 8 h	> 8 h

Table 3 depicts the characteristics of the 5 HCWs with a positive T-SPOT.*TB*. Results in absolute numbers from post-exposure screening of 155 health care workers (HCWs) and 48 healthy controls, 2005–2007. During childhood all participants were either BCG-immunized or had a naturally positive tuberculin skin test (TST). The definition of a positive TST (Mantoux) was an increase of ≥ 10 mm, or of ≥ 15 mm if previous TST status was unknown (in concordance with the national guidelines). The exposed HCWs were also tested with T-SPOT.*TB*, an interferon gamma release assay (IGRA).

All individuals detected as T-SPOT.*TB *positive belonged to the TST positive group, and the percentage concordance between T-SPOT.*TB *and TST, including both previously and newly infected subjects, was 12% (5/42). Two out of these five individuals were born in a TB high-endemic country. Thus, according to the T-SPOT.*TB *results the frequency of latent TB in the total cohort was 3% (5/155), whereas the TB transmission rate in the actual TB exposure study was estimated to be 2% (3/155). The 48 control participants were all T-SPOT.*TB *negative, but 3 persons in the control group were TST positive.

**Figure 1:**
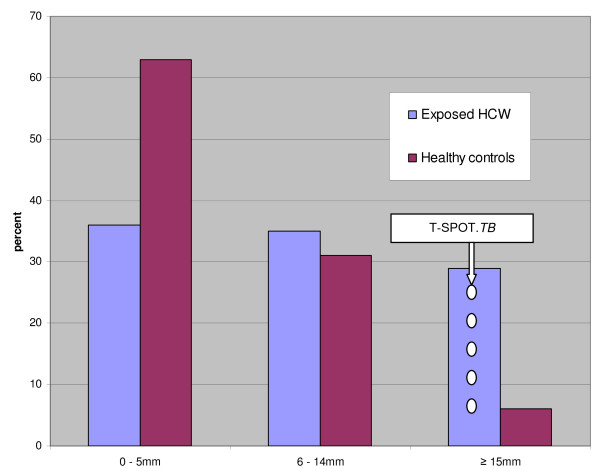
**Distribution of Mantoux test results by mm induration among 155 exposed health care workers and 48 healthy controls**. Left columns: percent of total number of exposed health care workers (HCWs). Right columns: percent of total number of healthy controls. HCWs with a previous positive TST (by Mantoux test ≥ 10 mm increase or ≥ 15 mm if previously unknown) where not retested, but were counted as ≥ 15 mm. The distribution of T-SPOT.*TB *positives is indicated by white circles; all of these 5 HCWs had ≥ 15 mm induration or were vesicular.

The distribution of TST results, given by mm induration, within the exposed and control group is given in Figure 1. A higher frequency of exposed HCWs with an induration of more than 14 mm was seen. A majority of the healthy controls had an induration of less than 6 mm.

The average time between first exposure and testing was 11.5 weeks. Among the exposed individuals, 51 participants belonged to the "high exposure" group (> 8 hours), and 104 participants fell into the "low exposure" group (≤ 8 hours) (Table 1). There was no correlation between length of exposure and TST results. In addition, there was no correlation between T-SPOT.*TB *positivity and TST results. For the 3 T-SPOT.*TB *positive individuals infected after known exposure, two were exposed ≤ 8 hours and one was exposed > 8 hours.

## Discussion and conclusion

A positive TST has previously been considered the gold standard in screening for *M. tuberculosis *infection. However, there is currently no definitive way to decide whether a person is latently infected with *M. tuberculosis*. A recent prevalence study by Soborg et al. in a Danish hospital confirmed earlier findings that TST is hampered by low specificity in BCG-vaccinated individuals [10,11]. The authors found a 34% prevalence among TST-positive HCWs, but the only significant risk factor associated with a positive TST was prior BCG immunisation; no association was observed with other important risk factors, such as occupational exposure to TB or hospital staff position [10]. Other low-endemic country studies have also found that a positive TST test is primarily associated with prior BCG vaccination and the boostering effect of prior TST testing [12-15]. Nevertheless, how can we conclude that the low post-exposure rate of infection detected by T-SPOT.*TB *in our study represents the real situation if there is no gold standard? One possible strategy is to estimate the likelihood of having latent TB infection by calculating a contact score that quantifies exposure to and infectiousness of the index case, as was done by Shams et al. [16]. A range of other studies also provide extensive evidence that the IGRAs correlate better to exposure than does TST. Therefore, we based our conclusions regarding the prevalence of TB infections on the T-SPOT.*TB *test [5,12-14,16-21].

Of 155 exposed HCWs and 48 healthy controls, all but one had a visible scar from BCG immunisation, which has been compulsory in Norway at the age of 14 until recently. Norwegian legislation also demands that all HCWs are asked for a certificate of TST at the time of appointment, and if the existence of a recent TST cannot be documented then a new TST is performed. The high correlation between BCG vaccination and a positive TST and the high specificity of the T-SPOT.*TB *test for *Mtb *infection (98%) make it likely that these are mostly false positive TST reactions rather than false negative T-SPOT.*TB *results. In addition, as found by Nienhaus et al. [14], the boosting effect of repetitive skin testing in health personnel may also contribute to the somewhat surprisingly low concordance between infection status according to T-SPOT.*TB *and TST that was observed in this study (12%). Still, there is no diagnostic gold standard for latent TB and the fact that 88% of employees recently defined as infected by TST tested negative with the T-SPOT.*TB *test calls for further studies of kinetics and immune mechanisms in TB infection. In our study all five T-SPOT.*TB*-positive individuals also had a strong positive TST. Nienhaus et all found that 5.1% of the HCWs in three German hospitals had a negative TST and a positive QFT [14]. While the combination of a negative IGRA and a positive TST is largely explained by prior BCG immunisation and TST boostering, the other combination is not readily explained, and further research is needed.

In contrast to most other studies related to TB transmission within health institutions, we have in this work compared the performance of T-SPOT.*TB *and TST in a group of HCWs with well-defined short-term exposure to contagious TB patients in a hospital setting. In addition, the results have been used to evaluate the role of IGRAs in improving the surveillance of TB transmission to health personnel in a low-incidence country like Norway.

Provided that the T-SPOT.*TB *results are the most reliable compared to TST results, our study indicates that the risk of infection among health care workers after short-term exposure to TB patients in a hospital setting is low (2%). This somewhat contradicts the findings of a Swiss long-term institutional study in which 15% of contacts were T-SPOT.*TB*-positive after prolonged unprotected exposure [13]. Although both studies were performed in health care institutions in low incidence countries, the exposure time may account for the observed differences in transmission. A study from Denmark also reported a low proportion (1%) of latent TB among HCWs as detected by the QFT test [10]. However, these data were not based on recent exposure, but rather represent the general prevalence level among hospital personnel working in departments with TB patients. We found a prevalence level of 3% in our cohort. Not surprisingly, these results are in contrast to findings from a high-endemic country like Russia, where a study utilizing QFT revealed a prevalence level of 41% among hospital staff working with infectious diseases [22]. Several reports based on TST conversion indicate that the risk of being infected may be high, even within a limited time frame of exposure [23,24]. This has also been confirmed by a T-SPOT.*TB *study in Italy in which 32% of the staff in a maternity ward became positive for TB after a mean exposure time of 6 hours [19]. Compared to these findings, our study detected a low degree of transmission. However, it should be noted that the majority of the individuals in this study had been exposed for less than 8 hours before precautions were taken. Still, among the three persons with positive T-SPOT.*TB *tests (believed to be recently infected), two were exposed for less than 8 hours. The absence of a statistically significant correlation between exposure time and both TST and T-SPOT.*TB *results is probably due to both the low exposure time and the small number of participants, since these correlations have been demonstrated in many other studies [19,23,24].

Importantly, the results indicate that using IGRAs as an alternative to the present follow-up strategy based on TST results could save substantial resources. Although TST by itself will be less expensive than a comprehensive laboratory procedure, the utilization of *Mtb*-specific blood tests has the potential to save major resources as the number of persons who must be followed up for 3 years can be reduced by up to 88%. In this context, the possibility of avoiding unnecessary and costly treatment, including serious side effects, is also of considerable importance. In addition, most exposed health care workers will avoid long-term anxiety by obtaining a negative result at a very early investigational stage. Finally, the small number of infected persons who require treatment can be identified immediately.

Oxlade et al. performed a 20-year cost-benefit analysis that used Markov modelling to compare the costs of TB screening with different strategies among hypothetical cohorts of foreign-born immigrants to Canada and contacts of TB cases. Model inputs were derived from published literature and utilization of the QFT test. For entering immigrants, screening with Chest X-Ray would be the most cost-effective and QFT the least cost-effective strategy. Sequential screening with TST followed by QFT was more cost-effective than either QFT or TST alone. In contact tracing after exposure, however, screening with TST followed by QFT, if positive, was more cost-effective than any other strategy. This was largely because TST alone was not effective if the exposed group had been vaccinated with BCG after infancy [25]. These findings were also confirmed by a Swiss study by Wrighton-Smith et al., estimating the costs of screening a cohort of 1000 individuals for latent tuberculosis; screening with TST alone followed by Chest X-Ray and clinical follow up of the positive cases was estimated to €695820; T-SPOT.*TB *alone was estimated to €387135; TST followed by T-SPOT.*TB *of the positives was estimated to €342563, i.e. the less costly [26].

Due to the fact that BCG immunization has been administered routinely in Norway, specific blood tests should be introduced in all post-exposure contact tracing situations. Because TB transmission to health personnel in Norway seems to be rather low, the two-step screening approach (TST followed by IGRA) might be attractive. However, some studies indicate an unacceptable low sensitivity of TST; Nienhaus et al. found that 40% of the HCW with latent TB infection according to IGRA results had a negative TST, and would have been missed utilizing TST followed by QFT of the TST positives. There is also evidence that a positive IGRA is a much better predictor of future reactivation than a positive TST [27], which is of particular interest because the Norwegian guidelines recommend prophylactic treatment in all documented cases of latent TB infection aged < 35 years. Further research is obviously needed to define improved screening strategies in low-endemic settings, both in the light of sensitivity and cost-effectiveness.

It should also be noted that the QFT method, although less sensitive in immuno-suppressed individuals, has both logistic and economic advantages compared to the T-SPOT.*TB *assay, and implementation of the QFT test has recently been suggested in Norway's national guidelines. The introduction of specific T-cell based assays for post-exposure screening and subsequent prophylactic treatment will become a rational and important component of the national TB control strategy.

## Competing interests

The authors declare that they have no competing interests.

## Authors' contributions

DGS initiated the study, participated in its design, interpretation of data and writing of the manuscript. IK and MG coordinated and supervised the practical implementation, took part in interpretation of data and writing of the manuscript. AKØ and GG coordinated the inclusion of participants at Ullevål University hospital and Haukeland University Hospital, respectively. FO and GEK took part in the design of the study, performed the T-SPOT.*TB *tests, interpretation of data and writing of the manuscript. GAB participated in the design, interpretation of data and writing of the manuscript. All authors read and approved the final manuscript.

## Pre-publication history

The pre-publication history for this paper can be accessed here:

http://www.biomedcentral.com/1471-2334/9/60/prepub
